# Changing Invisible Landscapes – Financial Reform of Health and Care Systems: Ten Issues to Consider

**DOI:** 10.5334/ijic.6461

**Published:** 2021-11-18

**Authors:** Liz Schroeder, Henry Cutler

**Affiliations:** 1 Centre for the Health Economy (MUCHE), Macquarie University, Sydney, NSW, Australia

**Keywords:** funding models, integrated care, health care reform

The COVID-19 pandemic has presented serious challenges to health care systems that were already under strain, and suffering their own chronic diseases of fragmentation, underfunding, siloed services, misaligned workforce configurations and overwhelmed hospital-centric activity. In many countries these cracks became craters forcing governments to self-reflect and to swiftly find new innovations in delivering care. *Never let a crisis go to waste*…

Globally in the years prior, health care was slowly transitioning from hospital-centric services to integrated care delivered in the community, but the groundswell of need from COVID-19 has re-prioritized person-centred services. Where governments were sluggish to enact substantial policy reform, COVID-19 has created a perfect storm and thus a greater obligation for change, including significant shifts in Treasury budgets to support welfare, in fiscal stimulus packages to support economies, policies to address the determinants of health and the provision of non-traditional forms of healthcare. In Australia greater supports have been provided for ‘hospital in the home’ services, where patients are able to receive high quality acute care without needing to be in hospital [1]. There has also been a much greater reliance on telehealth with GPs and specialists.

Countries operate their health care systems differently, but many suffer from common problems, such as too little clinical care delivered in the community, leading to an overreliance on acute inpatient care. Transitions from siloed funding to newer funding approaches that support the integration of care require provider incentives to align. Different funding models create different financial incentives, which in turn lead to different services being offered and accessed. Quite simply, funding models can impact access to health care, and therefore health outcomes, in a substantial way.

## Policy levers in health care

To that effect, we can assign funding models to the same camp as policy levers, enablers to address market failures and agency relationships in healthcare. Funding model reforms often shift funding from one provider to another, or funding risk from payer to provider. Funding models are not silver bullets and should only exist within their remit as one of the nine pillars of integrated care [2], and in many circumstances other policy levers may be preferred to secure quality healthcare improvement [3,4]. Furthermore, funding models carry inherent risk in that they are an indirect approach to changing behaviour, and therefore open to unintended consequences. Prior to commencing funding model reform, it is worth considering whether an alternative policy lever would be better. For example, improving quality may be better addressed by improving a performance management approach rather than introducing financial incentives to change behaviour.

That said, funding models can introduce financial incentives to motivate provider behaviour to improve health care quality and efficiency. They are typically implemented within a suite of measures and often contractual obligations, such as performance reporting, to promote policy objectives. Thus, they can be used to encourage service providers to deliver a quantum and mix of services aligned to government policy or patient preferences.

No clear ‘best’ funding model exists that operates within health care, though certain models are better suited for funding certain types of services. For example, in the case of mental health – the US mostly funds care through private health insurance provided by employers, which use a variety of funding models, such as capitation and fee for service. There is also a broader push within the US health care system to deliver value-based care, which is being encouraged by replacing fee for service funding with alternative payment models that reward increased value [5].

This can be compared to alternative funding model approaches in England, where mental health care is publicly funded through the NHS, with around 70 per cent of funding administered through the 135 clinical commissioning groups (CCGs). A shift away from block funding towards episodic payments has occurred, along with some CCGs exploring a shared funding risk model with providers through accountable care systems (ACSs) and capitation-based payment models [6]. GPs are funded through the Quality and Outcomes Framework, which rewards GPs for meeting care management and preventative services targets across eleven mental health indicators.

## Selecting a funding model to underpin financial incentives for integrated care

Any health care funding model should account for the unique care environment, organisational structures, governance arrangements and legacy funding arrangements. Genuine funding reform cannot replace investment into health care, such as an appropriately trained and available workforce, and up to date investments in technology, data platforms, health infrastructure and medical products.

All funding models have their advantages and disadvantages and selecting a funding model to achieve a strategic objective will require trading off one important criterion for another. Different governments will have different criteria, and of the same criteria, governments may weigh the importance of each criterion differently. For example, an extraction of funding model criteria from twelve Australian policy documents on public hospital funding resulted in 32 potential criteria. Defining the common themes led to 15 funding model criteria which included both similar and distinct preferences, such as continuity of care, data needs, choice, effectiveness, risk minimisation, simplicity, or responsiveness [7].

Selecting a funding model typically requires a trade-off between funding model complexity and the ability to incentivise good quality care. More complex funding models require more high-quality data. Bundled payments and capitation require data for costs and resource use to calculate prices, patient characteristics to risk adjust funding, and on outcomes to reward providers for good practice. This requires investment in data collection and analysis. Simpler models, such as Historic Block Funding or transactional Fee for Service do not depend on algorithms to underpin funding arrangements but deliver poor incentives to improve quality on their own.

Genuine funding model reform should be developed around an agreed set of principles, align with broader healthcare system priorities (e.g., value based or integrated care), incentivize the efficient delivery of evidence-based care, allow innovation to flourish and align with reforms proposed for other sectors of health care. New approaches for system integration will also explore how services previously provided through hospitals can be substituted in the community, and the capability of fund holders to manage them [8]. Funding models can improve value when optimally designed within a specific health care context, though effectiveness will likely depend on the interrelationship between design characteristics, governance structures, infrastructure, and culture.

Structural reform requires cycles of monitoring and review for improvement, and key attributes in the process should include engagement with stakeholders, measurement of outcomes, an assessment of the viability of change and the creation of an enabling environment to support reforms to mature.

## Changing the Landscape

We propose ten generic *issues to consider* when contemplating healthcare system reform towards more integrated care, which would also require iterative, nuanced, and thoughtful application to local context.

- Develop a principles framework for funding reform to guide the process of change.
- Commence funding model reform by developing clear objectives towards the provider behaviours that should change, be mindful of the financial context and governance structure, objectives and capacity of providers, and constraints of the system (e.g., workforce).
- Consider the new incentive structure and purpose; identify where the design of the new models will create effective and efficient funding flows.
- Review legislative local and regional governance in view of new aspirations. Design governance frameworks for funding models that ensure transparency and responsibility in arrangements and care continuums. Explore where arrangements in governance may produce perverse incentives.
- Identify persisting service and integration gaps; and real-time assessment of community services and supports.
- Invest in linked and individual patient identifier data that could inform complex models for delivering integrated healthcare e.g. bundling or case-mix capitation requires granular data to inform costs, resource use classification from prior utilisation patterns to predict pricing, data linkage and clinical consensus of best practice. Accurate predictive modelling would be needed to create normative capitation pricing mechanisms, and to evaluate these for a risk of over or under-servicing.
- Develop mechanisms to evaluate for cost-effectiveness, opportunity cost, system integration, viability, and scalability.
- Collect consistent, whole of system health outcomes data that could progress reforms into the next stage, towards value-based care that combines investment with health outcomes instead of activity. Include where possible, opportunities to collect Patient Reported Measures (PRMs), evidence-informed care and clinical perspectives.
- Test innovations and new schemes using pilot, phased roll-out to scale or shadow pricing approaches.
- Where possible, adapt funding models and flows to incorporate a proactive lens to resolving equity issues and social determinants.

Funding model reforms are extremely challenging, having to address intractable and wicked problems of complex system redesign and imbedded incentives. Clear leadership is required to guide and champion a coherent design of the building blocks for reform, across all levels in a staged and iterative approach [9]. Providers should be given support to improve quality through staffing, infrastructure, team functioning and use of quality improvement tools [10]. A workforce needs to be trained to provide community delivered integrated services.

Structural reforms need to outlive economic and political cycles while providing stability during prolonged periods of transitions in service delivery, as the system realigns itself towards a new funding model. The reforms may take many years to embed, and consequently perhaps the greatest risk to a ‘post-Covid world’ is an exchange of short-term gains for longer-term reform.

Longer term reform requires time to invest to reconfigure the services and infrastructure. Realignment will require the ongoing monitoring of progress, and evaluation for system adjustment and maturity. Evaluation should focus on health outcomes, cost effectiveness, and unintended consequences. It should allow for potential learning over the adoption phase and identify potential areas for further improvement. Through these processes, stakeholders at all levels will need to invest patiently for the progressive realisation of healthcare that is high quality, integrated and person-centred.
